# Effect of a mouthrinse containing rice peptide CL(14-25) on early dental plaque regrowth: a randomized crossover pilot study

**DOI:** 10.1186/s13104-015-1527-8

**Published:** 2015-10-03

**Authors:** Saori Takayama, Tetsuo Kato, Kentaro Imamura, Daichi Kita, Koki Ota, Eiichi Suzuki, Hiroki Sugito, Eiichi Saitoh, Masayuki Taniguchi, Atsushi Saito

**Affiliations:** Department of Periodontology, Tokyo Dental College, Tokyo, Japan; Laboratory of Chemistry, Tokyo Dental College, Tokyo, Japan; Oral Health Science Center, Tokyo Dental College, Tokyo, Japan; Department of Endodontics and Clinical Cariology, Tokyo Dental College, Tokyo, Japan; Graduate School of Technology, Niigata Institute of Technology, Niigata, Japan; Department of Materials Science and Technology, Graduate School of Science and Technology, Niigata University, Niigata, Japan; Center for Transdisciplinary Research, Niigata University, Niigata, Japan

**Keywords:** Periodontal disease, Mouthrinse, Rice peptide, Antimicrobial agents, Plaque, Biofilm

## Abstract

**Background:**

We aimed to evaluate clinically the effect of mouthrinse containing a rice peptide on early dental plaque regrowth.

**Methods:**

The study was designed as a double-masked, two-group crossover randomized pilot trial, involving 10 periodontally healthy volunteers. After receiving a professional tooth cleaning at baseline, over the next 3 days each participant refrained from all oral hygiene measures and had two daily rinses with 20 ml of the test mouthrinse containing 0.4 % rice peptide CL(14-25) or placebo rinse. At the end of each experimental period, plaque score was assessed using the modified Volpe’s method, and the participants filled out a questionnaire. Each participant underwent a 7-day washout period followed by a second allocation. The plaque score was the primary outcome of the study and participant perception was the secondary outcome.

**Results:**

No adverse effects were observed in the participants during the study. Clinically, the mean plaque score of the examined teeth was significantly lower in the test group (2.44 ± 0.74, CI: 1.91–2.96) than the placebo group (2.65 ± 0.63, CI: 2.20–3.10) (*P* < 0.05). When analyzed according to the type of teeth, a significantly lower score of the premolars/molars was observed in the test group (2.39 ± 0.68, CI: 2.08–2.71) than that in the placebo group (2.66 ± 0.58, CI: 2.39–2.93) (*P* < 0.05).

**Conclusions:**

The mouthrinse containing 0.4 % rice peptide CL(14-25) was effective in reducing the early regrowth of dental plaque. However, clinical relevance of this efficacy needs to be validated in a future large-scale study.

Trial registration: UMIN Clinical Trials Registry (UMIN-CTR) R000014000. Date of formal registration: November 1, 2013.

## Background

Periodontal disease is the inflammation of periodontal tissue caused by pathogenic microflora in dental plaque biofilm [1]. Theoretically, it is possible to prevent plaque-induced periodontal disease by meticulous mechanical oral hygiene. However, many individuals have difficulty in maintaining the necessary standards of dental plaque control for prolonged periods [2], and the periodontal disease remains to be one of the most prevalent diseases of humans.

In order to control plaque biofilms, numerous antiplaque and antimicrobial agents have been formulated into toothpastes and mouthrinses [2–5]. The efficacy of those agents used in adjunct to mechanical plaque control has been demonstrated in many studies [6–8]. However, the antimicrobial agents could disrupt the natural microbial ecology of the mouth, which might result in overgrowth of opportunistic or resistant pathogens [2]. Ideally, an antimicrobial agent should not induce drug-resistance or disrupt the oral microbial ecology.

Some of the natural products, particularly antimicrobial peptides, have received increased attention as promising antimicrobial agents [9]. CL(14-25), a dodecapeptide, that is a partial region near N-terminus of cyanate lyase (CL, EC 4.3.99.1, GenBank ID: Os10g0471300) from rice (*Oryza sativa* L. spp. *japonica*), is a novel cationic α helical antimicrobial peptide with three arginine and two lysine residues [10]. CL(14-25) has been shown to inhibit the growth and activity of a prominent periodontal pathogen, *Porphyromonas gingivalis* [11, 12]. In our recent in vitro experiments, the CL(14-25) demonstrated neutralizing activity against endotoxin of *Aggregatibacter actinomycetemcomitans* [13] and reduced the biofilm formation by *P. gingivalis* in a dose-dependent manner (unpublished observation). We also found that co-incubation of CL(14-25) (0.07–0.35 mM) for 4 and 24 h induced no significant decrease in the viability of human aortic endothelial cells. Following the gavage administration of 0.2 mg CL(14-25) preparation/mouse to BALB/c mice, no significant difference in change in body weight was found after 14 days compared to control mice. No significant change in behavior or fur coat condition was observed (unpublished observation). These findings lead us to hypothesize that the CL(14-25) may be useful for the control of dental plaque biofilm.

The aim of this pilot study was to evaluate clinically the antiplaque effect of CL(14-25) in mouthrinse formulation, using an in vivo dental plaque regrowth model of 3-days.

## Methods

This study was approved by the ethics committee of Tokyo Dental College (No. 386).

### Peptides

The amino acid sequences and properties of CL(14-25) used in this study are summarized in Table 1. The sequence of CL(14-25) is identical to that of CH peptide reported by Taiyoji et al. [10]. Chemically synthesized CL(14-25) was obtained from Hokkaido System Science Co., Ltd. (Sapporo, Japan). Synthetic peptides were purified to >95 % by reversed-phase high-performance liquid chromatography (RP-HPLC). Molecular weights of purified CL peptides were confirmed by matrix-assisted laser/desorption ionization–time-of-flight mass spectroscopy (MALDI–TOF-MS).

**Table 1:** The amino acid sequence and properties of CL(14-25)

Sequence	RRLMAAKAESRK
Size [a.a.]	12
Net charge	+4
Molecular mass^a^	1416.72

^a^The values indicate molecular mass of each purified peptide confirmed by MALDI–TOF analysis

### Study population

Participants were recruited from periodontally healthy individuals, between November 2013 and December 2013. Written informed consent was obtained from all participants. To qualify, subjects were required to have a minimum of 20 natural teeth. Systemic exclusion criteria were the presence of cardiovascular and respiratory diseases, systemic inflammatory conditions, such as diabetes mellitus, immunodeficiency, and current pregnancy or lactation. Current smokers and those with history of allergic reactions to rice, several mouthrinse components or medications were also excluded. The participants received no medication that could affect their periodontal conditions, such as antimicrobial agents or anti-inflammatory drugs, for at least 3 months prior to the microbiological testing.

### Mouthrinse formulation and preparation

Two formulations were compared. Test mouthrinse was prepared with 0.4 % CL(14-25) and 99.6 % sterilized distilled water [CL(14-25) 4.0 mg/ml]. No flavorants or preservatives were added. Distilled water was used as control (placebo) rinse. Preservation condition of the mouthrinse formulations was assessed by a standard microbial limit test using spread plate method according to the method specified in the Japanese Pharmacopoeia. It was found that the mouthrinse formulations can be safely stored at both 4 °C and room temperature during the study period.

### Dental plaque regrowth study

The main outcome variable was plaque score assessed by the modified Volpe’s method [14, 15] reported by Suzuki et al. [16]. After disclosing the plaque, a periodontal probe was used to measure the height of the plaque accumulation from the gingival margin to the nearest 0.5 mm at six sites per Ramfjord index teeth (#16, 21, 24, 36, 41, 44) [17] considering both buccal and lingual surfaces. Clinical examiners were trained in study procedures prior to commencement of the study. The plaque score of a given tooth was expressed as the absolute value from the sum of all six measurements [18].

**Fig. 1:**
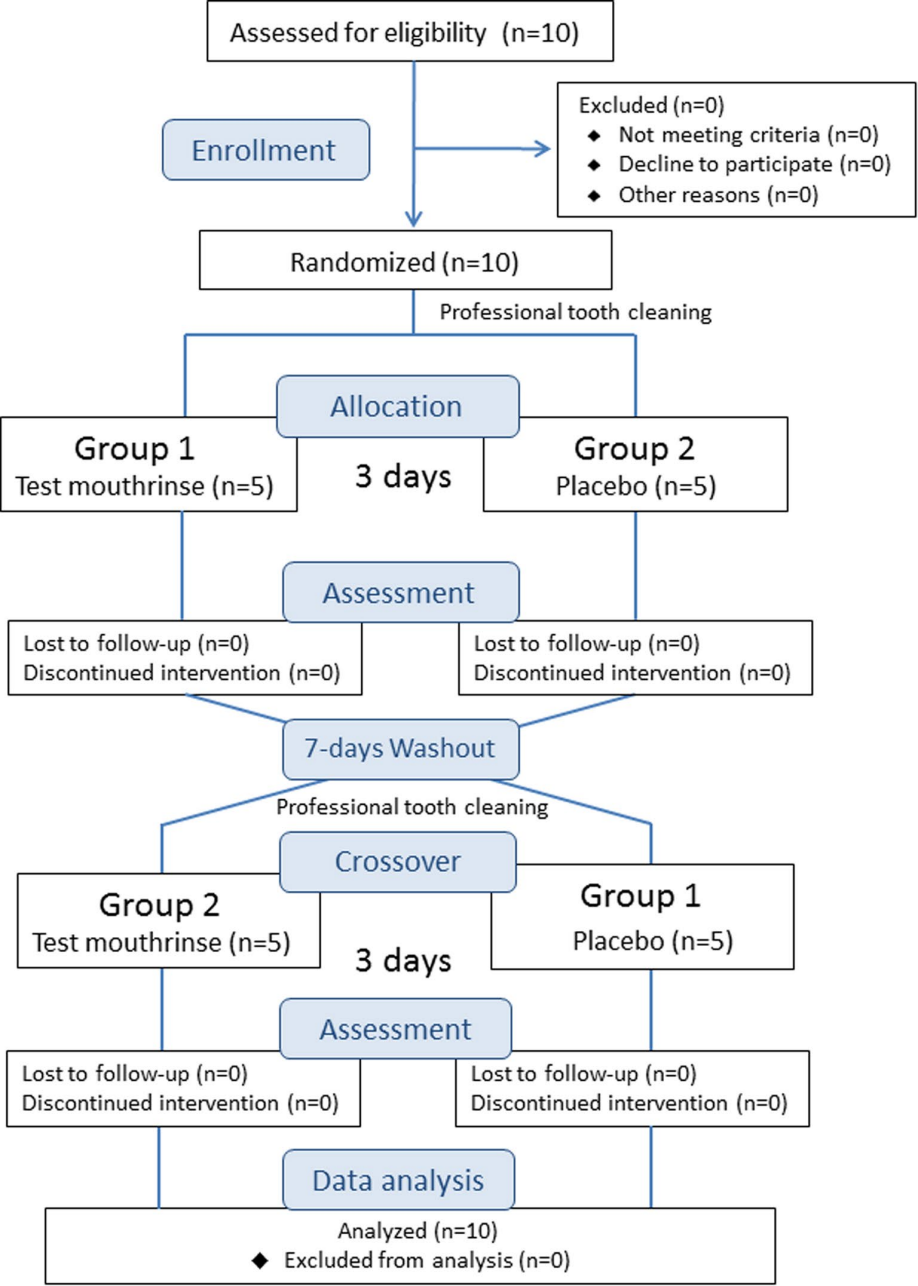
Flow diagram illustrating the study design

In Fig. 1, flow diagram of the study is presented. Within 7 days of the screening examination, eligible participants received oral hygiene instructions and professional tooth cleaning in order to standardize oral hygiene procedures. Through computer-aided randomization, the participants were divided in two groups of the same size; Group 1 and Group 2. A person not directly involved in the research project carried out the allocation of test or control products. A plaque regrowth study design, with a crossover design [19, 20], was used. The selected mouthrinse formulation was given to participants in Group 1 and the placebo mouthrinse was given to those in Group 2, along with the usage instructions. Each participant received a bottle containing 125 ml of mouthrinse. They were instructed to rinse their mouth with 20 ml of the given mouthrinse for 1 min twice a day for 3 days avoiding any other oral hygiene manoeuvre. To check for compliance, subjects were asked to note the times of day when they rinsed.

Study participants were asked to fill out a questionnaire regarding their perception of plaque reduction. Perceived plaque reduction was graded on a 5-point rating scale with discrete responses, ranging from 0 (insufficient) to 5 (very sufficient). At the completion of the trial, participants were asked to choose the mouthrinse formulation with which greater plaque reduction was perceived. All returned mouthrinse bottles were weighed to calculate the amount of mouth rinse used and to check for compliance.

After this period, plaque score was recorded using a disclosing solution. A washout period of 7 days separated each treatment phase. Then participants resumed their normal oral self-care. After this washout period, all participants underwent again a session of professional tooth cleaning and the protocol continued inverting the mouthrinses according to a crossover design and with the same timing and mode of use previously described. In this way, each subject received both treatments sequentially. Plaque reduction rate was calculated as follows: Plaque reduction (%) = [(plaque score of the placebo rinse) − (plaque score of the test rinse)/(plaque score of the placebo rinse)] × 100.

### Statistical analysis

The tooth was the unit of analysis. Data were analyzed using nonparametric statistics, which are more appropriate when the data show a skewed distribution. Thus, the Wilcoxon matched-pairs signed-ranks test was used to ascertain the differences between the individual rinse solutions. Data considering the questionnaire scores were also analyzed using the same test. Fisher’s exact test was used to compare the association with the two different score category between groups.

All data were registered electronically using Microsoft Excel, and statistical analyses were carried out using a statistical package (InStat 3.10, GraphPad Software, La Jolla, CA, USA). *P* values less than 0.05 were considered as statistically significant.

## Results

### Compliance and adverse events

Ten eligible volunteers (all males; age range 26 to 33 years; mean age: 28.2) participated in the study. At baseline, the mean total plaque score of the examined teeth of the participants was 1.18 ± 0.97, as assessed by the scoring method by Suzuki et al. [16]. All participants (N = 10) completed the trial, and there were no missing values.

The amounts of mouthrinses used indicated good compliance with the instructions. There were no significant adverse events, including mucosal irritation, tooth staining and taste alteration in relation to the use of the mouthrinse formulations.

### Effects on the plaque score

**Fig. 2:**
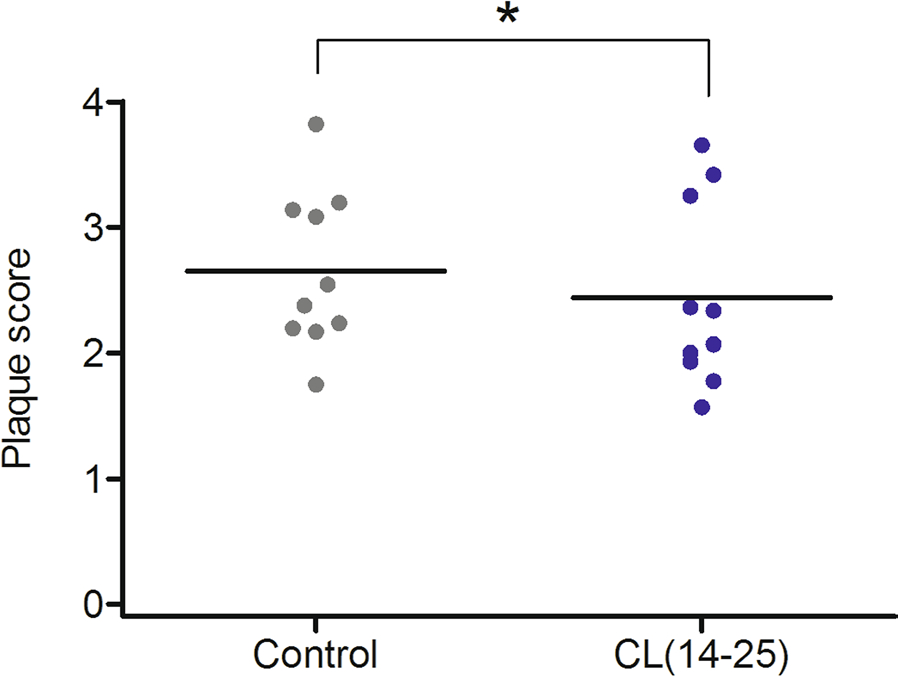
Plaque scores of the teeth following the use of control (placebo) or test mouthrinse: all teeth examined. The *line* represents mean value. (*P < 0.05, Wilcoxon matched-pairs signed-ranks test)

**Fig. 3:**
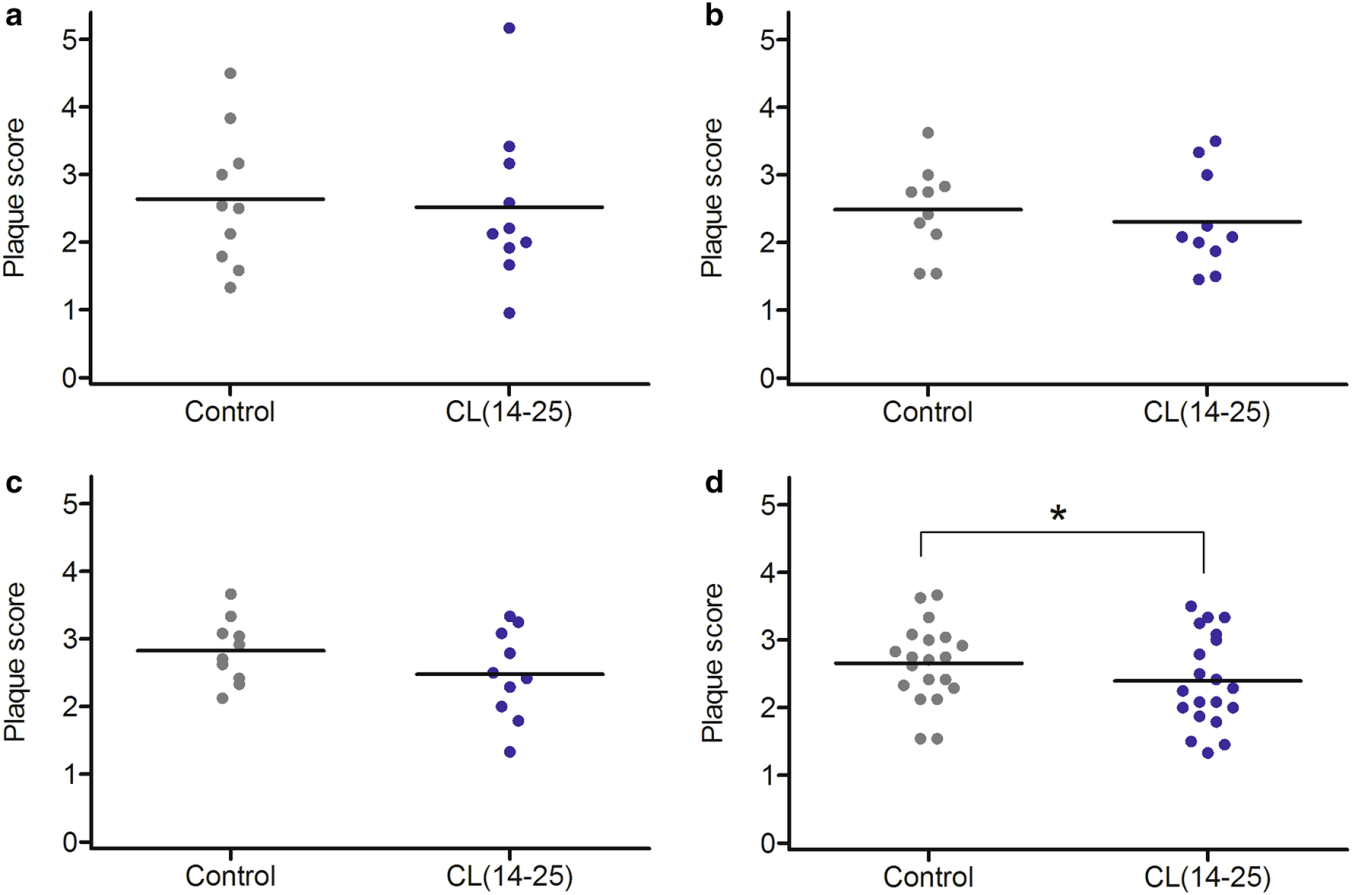
Plaque scores of the teeth following the use of control or test mouthrinse: analysis by the tooth type. **a** incisors, **b** premolars, **c** molars, **d** premolars/molars. The *line* represents mean value. (*P < 0.05, Wilcoxon matched-pairs signed-ranks test)

The plaque scores of the teeth examined after the use of test or control mouthrinse are shown in Fig. 2. There was significant difference in plaque score between the CL(14-25) group (2.44 ± 0.74, CI: 1.91–2.96) and the placebo group (2.65 ± 0.63, CI: 2.20–3.10) (*P* < 0.05). When analyzed according to the types of teeth (Fig. 3), a significantly lower mean plaque score was observed in the premolars/molars in the CL(14-25) group (2.39 ± 0.68, CI: 2.08–2.71) than the placebo group (2.66 ± 0.58, CI: 2.39–2.93) (*P* < 0.05) (Fig. 3d).

The mean plaque reduction rate (relative to control rinse) of the examined teeth was 8.8 ± 10.4 % (Table 2). When analyzed according to tooth surface of the examined teeth group, the plaque reduction rate was the greatest (19.7 ± 21.9 %) on the lingual surfaces of molars. In contrast, the mean plaque reduction rate was low for incisors (4.2 ± 22.2 %). Especially, in lower incisors and on lingual surface of incisors, there was no improvement in values.

**Table 2:** Plaque reduction (%)

	Total	Upper	Lower	Buccal	Lingual
Incisor	4.2 ± 22.2 (−11.7 to 20.1)	11.0 ± 32.7 (−12.4 to 34.4)	−4.4 ± 26.0 (−23.1 to 14.2)	3.5 ± 36.4 (−22.5 to 29.6)	−1.4 ± 36.6 (−27.5 to 24.8)
Molar	12.5 ± 17.4 (0.1 to 30.0)	13.8 ± 17.1 (1.6 to 26.1)	9.3 ± 26.0 (−9.3 to 27.9)	7.0 ± 19.5 (−7.0 to 20.9)	19.7 ± 21.9 (4.0 to 35.4)
Premolar	7.0 ± 15.2 (−3.9 to 17.9)	6.4 ± 18.7 (−7.0 to 19.8)	8.8 ± 20.5 (−5.8 to 23.5)	3.2 ± 15.9 (−8.2 to 14.5)	8.9 ± 29.5 (−12.2 to 30.0)
Molar and premolar	9.8 ± 16.2 (2.2 to 17.3)	10.1 ± 17.9 (1.7 to 18.5)	9.1 ± 22.8 (−1.6 to 19.7)	5.1 ± 17.4 (−3.1 to 13.2)	14.9 ± 25.9 (−2.2 to 26.4)
All teeth examined	8.8 ± 10.4 (1.3 to 16.2)	11.6 ± 12.8 (2.5 to 20.8)	5.7 ± 17.6 (−6.9 to 18.3)	6.7 ± 17.8 (−1.6 to 15.0)	9.2 ± 23.5 (−1.8 to 20.2)

Plaque reduction (%) = [(plaque score of the placebo rinse) − (plaque score of the test rinse)/(plaque score of the placebo rinse)] × 100. Data shown as means ± standard deviations (lower 95 % confidence interval − upper 95 % confidence interval)

### Participants’ perception of plaque reduction

With regard to the perception of plaque reduction, no significant difference in score was observed (mean scores: 2.4 for the test rinse and 2.3 for the control). The number of participants who gave scores higher or equal to 3 was 5 in the test group whereas that in the control group was 3 (no significant difference, *P* = 0.650, Fisher’s exact test). As for the overall perception of plaque reduction comparing two different formulations at the completion of the trial, three participants rated the test rinse better than the control rinse and seven rated them as the same.

## Discussion

In order to overcome the problems associated with the use of conventional antimicrobial agents in oral rinse formulations, novel natural antimicrobial ingredients have been studied [21–24]. However, evidence providing the effectiveness of natural compounds containing mouthrinse as an adjunct to unsupervised oral hygiene for the control of dental plaque is still insufficient [25]. In previous studies from our research group, we have shown in vitro the various antimicrobial properties of the CL(14-25), a deodecapeptide derived from rice [10–13]. Based on these findings, we evaluated the antiplaque effect of the CL(14-25) in mouthrinse formulation in this study. The data demonstrated that rinsing with a 0.4 % CL(14-25) solution resulted in a significant decrease in the plaque score of the teeth examined. This finding may be particularly pertinent in view of the growing interest in the use of natural ingredients in healthcare products.

We used a non-brushing model that allowed for dental plaque accumulation that has been widely used to evaluate the effects of various mouthrinses [19–22, 26–28]. Conducted in the absence of mechanical oral hygiene, the method provides direct evidence of the chemical action of formulations against plaque growth [29]. Using this study design, we have shown that the mean plaque reduction rate of the examined teeth was 8.8 %. This value is greater than that (~1.9 %) reported with 0.03 % sanguinaria extract [29] but is lower than those reported with mouthrinses containing essential oil or chlorhexidine (reduction rates; 17–38 %) [26–30]. It is difficult to directly compare these results, as the evaluation methods of plaque accumulation are different. Also, the potential difference in the ability to form plaque biofilm between participants of different studies may have influenced the outcome. Given that no other ingredients were added to the CL(14-25) mouthrinse formulations, the data suggest its clinical efficacy in reducing the regrowth of dental plaque. The mechanisms for the reduction in plaque regrowth are thought to involve the antimicrobial properties of the CL(14-25) [11–13], including those against periodontal pathogens. We speculate the reduced viability of the biofilm organisms following the use of the CL(14-25) mouthrinse.

In this study, we used the plaque scoring method by Suzuki et al. [16], which evaluates the extent of dental plaque formation on tooth surface. This scoring method has been used in various clinical trials [18, 31–33]. Keeping the difference of scoring methods in mind, the finding that relatively high plaque reduction rate (19.7 %) was noted for the lingual surface of molars is similar to that by Ramberg et al. [34]. Although the exact reasons for the seemingly superior reduction score for the lingual surfaces of molars are unclear, this finding may be clinically relevant since such region is generally more difficult to obtain satisfactory results by mechanical plaque control. In contrast, the plaque reduction was minimal for incisors, especially lower incisor and lingual surface. Similar finding was reported by Pizzo et al. [35, 36]. It may be argued that the lingual surfaces of lower incisors may not be adequate in evaluating the plaque reduction by the use of mouthrinse, since they are in close contact with the tip of tongue, which may influence the early plaque regrowth.

No significant difference in participants’ perception of plaque reduction was found between groups. It should be noted that three individuals rated the CL(14-25) rinse better than the placebo rinse in perceived plaque reduction, although the remaining seven participants rated both rinses as the same. We need to further investigate the subjective measure of the effect of the CL(14-25) mouthrinse, after adding other ingredients necessary for the commercial formulation.

It has been reported that the essential oil-containing mouthrinse, when used in conjunction with a fluoride dentifrice and usual oral hygiene, provided a greater benefit in reducing plaque [37]. This may hold true for the present CL(14-25) mouthrinse. This needs to be verified with studies incorporating mechanical plaque control measures. It is necessary for the antimicrobial agents not to disrupt the natural microbial ecology of the mouth, which might result in overgrowth of opportunistic or resistant pathogens. The long-term effects on the microbial ecology and on the periodontal tissues need to be investigated.

There are several limitations to our study. The sample size was very small and only male participants were included. The use of only the index teeth for assessment is another limitation. The choice of plaque scoring method made it difficult to compare the results with those reported in the previous reports outside Japan. This study is a pilot in nature and provides a framework upon which future studies can be based. Further studies employing larger sample sizes are necessary to fully elucidate the clinical effect(s) of the CL(14-25) on the control of dental plaque. Also, it is necessary to test the CL(14-25) in relation to other ingredients that can be formulated into mouthrinse.

Rice is the staple food in Japan and many other countries, and people’s general perceptions of this food are very favorable. It is relatively affordable, which is important in considering the ingredient for mouthrinse formulations. Our findings open up new possibilities for rice, in addition to its established role as an excellent dietary energy source.

## Conclusions

Our data indicate that the CL(14-25) has potential as an anti-plaque therapeutic product for oral use and warrants further clinical evaluation. The peptide and the formulation appear safe and well-tolerated by experimental participants.

## Abbreviations

CL: cyanate lyase
MALDI–TOF–MS: matrix-assisted laser/desorption ionization–time-of-flight mass spectroscopy
CI: confidence interval
